# Pediatric Liver Abscess: Trends in the Incidence, Etiology, and Outcomes Based on 20-Years of Experience at a Tertiary Center

**DOI:** 10.3389/fped.2020.00111

**Published:** 2020-03-24

**Authors:** Pai-Jui Yeh, Chien-Chang Chen, Ming-Wei Lai, Hung-Yu Yeh, Hsun-Chin Chao

**Affiliations:** ^1^Division of Pediatric Gastroenterology, Department of Pediatrics, Chang Gung Children's Medical Center, Chang Gung Memorial Hospital, Taoyuan City, Taiwan; ^2^Department of Medicine, Chang Gung University College of Medicine, Taoyuan City, Taiwan

**Keywords:** liver abscess, trends, *Klebsiella pneumoniae*, children, pyogenic

## Abstract

**Background:** Liver abscess is an important but relatively rare disease in children. This study investigated the clinical characteristics of eligible patients at a referral tertiary center over the past two decades.

**Method:** A 20-year retrospective study (January 2000–December 2019) enrolled 38 children diagnosed with liver abscess. Demographic data; clinical features; laboratory, imaging, and microbiological findings; management strategy; and outcomes were reviewed from the patients' medical records.

**Results:** Thirty-eight cases of pyogenic liver abscess were identified without a culture-proven amebic or fungal abscess. The mean age of diagnosis was 9.6 ± 6.2 years, and the male-to-female ratio was 1.92: 1. Hemato-oncological (28.9%) and predisposing hepatobiliary diseases (23.7%) were the two most common predisposing factors. Fever (94.7%) was the most common presentation followed by right upper quadrant abdominal pain (42.1%) and pleural effusion (34.2%). Among the laboratory parameters, leukocytosis was common (70.3%), and all patients had elevated serum C-reactive protein levels. Increased serum levels of alanine aminotransferase, aspartate aminotransferase, direct bilirubin, and total bilirubin were found in 40.5, 48.6, 23.1, and 42.9% of the cases, respectively. The most common pathogen in blood and pus cultures was *Klebsiella pneumoniae*. The mean durations of intravenous antibiotic and total antibiotic use were 29.0 ± 15.7 and 45.1 ± 22.1 days, respectively. Twelve patients (31.6%) were treated with antibiotics alone, while percutaneous needle aspiration, percutaneous pigtail drainage, and surgical intervention were performed in 12 (31.6%), 10 (26.3%), and 5 (13.2%) patients, respectively. No mortality was documented in this series.

**Conclusion:** The present study reflects a relatively declining incidence of liver abscess compared with prior studies in Taiwan. *K*. *pneumoniae* remains the most prevalent pathogen in both blood and abscess cultures in Taiwan. Proper antimicrobial therapy with timely drainage generally yielded an adequate treatment response without any mortality.

## Introduction

Liver abscess is an important yet relatively uncommon disease in children, with varied incidence and prevalence worldwide (1). Liver abscess is uncommon in developed countries but remains a significant infectious issue for children residing in developing countries (2–4). The incidence rates reported in previous studies ranged from 25 per 100,000 pediatric admissions in the USA, 79 per 100,000 pediatric admissions in India, and up to 400 per 100,000 pediatric admissions in Cote d'Ivoire (3, 5, 6). Although a nationwide all-age analysis for pyogenic liver abscess in Taiwan revealed an increasing annual incidence from 1996 (11.15 per 100,000) to 2004 (17.59 per 100,000) (7), two pediatric single institution-based studies disclosed a decline from 20 per 100,000 pediatric admissions in 1979–1992 to 8.3 per 100,000 pediatric admissions in 1986–2001 (8, 9). Subsequent analyses of the incidence of liver abscess in Taiwanese children is lacking in past decades.

The demographics and clinical features of liver abscess may differ among regions. For example, previous studies disclosed the dominance of *Klebsiella pneumoniae* as the causative pathogen in Taiwanese adults and children (2, 9, 10), while *Staphylococcus aureus* and *Entamoeba histolytica* are more prevalent in other countries. Mean age at diagnosis for Taiwanese children is relatively older than that in other series (3, 9–11). Overall, the treatment outcome has been improving gradually with higher clinical suspicion and rigorous treatment, although we still possess no consensual algorithm. The mortality rate due to liver abscess has dropped from 40% in the 1980s to below 15% (2, 4, 11).

Our study analyzed the clinical presentation, diagnostic approach, pathogens, treatment strategy, and outcome of pediatric liver abscess based on 20-years of experience at a single tertiary center.

## Materials and Methods

### Study Design

This was a retrospective study based on a review of medical records, enrolling pediatric patients (aged < 18 years old) who were diagnosed with a pyogenic liver abscess and hospitalized at Chang Gung Memorial Hospital Linkou branch between January 2000 and December 2019. All eligible cases received regular treatment and follow-up. Patients with incomplete medical records and uncertain diagnoses were excluded.

Liver abscess was defined by any of the following criteria: (1) hepatic lesion with typical image findings, associated with either a response to antimicrobial therapy or a positive blood or abscess culture; (2) positive culture from needle aspirated abscess; or (3) surgically proven abscess (9, 10). The diagnosis was established with the collaborated teamwork of a pediatric gastroenterologist, radiologist, pathologist, surgeon, and infectious disease specialist.

### Diagnostic Approach and Treatment Strategy

Abdominal ultrasonography is the first-line diagnostic tool for a liver abscess at our institution. Typical liver abscesses present with acoustic enhancement, an abscess wall, a peripheral halo, septation, and internal debris (12, 13). Computed tomography (CT) or magnetic resonance imaging (MRI) was performed as a secondary modality to confirm if the ultrasonographic findings were inconclusive for highly suspicious cases. In addition to basic laboratory examination and blood culture, abscess culture with antimicrobial resistance profile was obtained if feasible. All patients received antibiotic therapy for their abscesses. Further percutaneous aspiration (or drainage) or open surgery was required if the patient was in poor clinical condition or responded poorly to antibiotic therapy.

### Antibiotic Treatment

The routine first-line parenteral antibiotics for liver abscess were ampicillin or a first-generation cephalosporin plus gentamicin and metronidazole. Second-line parenteral antibiotic regimens contained a third-generation cephalosporin. Imipenam or piperacillin-tazobactam was used for advanced conditions. Antibiotic escalation was considered in patients whose peripheral white blood cell (WBC) count, band-cell ratio, or serum C-reactive protein (CRP) level did not improve within 3 days of the initial antibiotic treatment.

### Data Collection and Analyses

Data collected from the medical records included demographic information, duration of symptoms, laboratory values (peripheral WBC counts, neutrophil counts, band-cell ratio, serum CRP level, blood, or abscess culture results), imaging findings, categories of initial intravenous (IV) antibiotics, duration of IV and total (IV plus per-oral) antibiotic treatment, need for interventional treatment (percutaneous aspiration, percutaneous drainage, or open surgery), length of hospitalization, complications, recurrence, and mortality. Mortality and recurrence were assessed based on documentation in the chart, including any available information on subsequent admissions or outpatient visits. Time to resolution of the liver abscess and duration of hospitalization were also recorded.

### Statistical Analyses

Data entry and processing were conducted using the Statistical Package for the Social Sciences (SPSS) v. 20 software (SPSS Inc., Armonk, NY, USA). Continuous variables are expressed as means ± standard deviations or as medians with interquartile range as appropriate and categorical variables are expressed as percentages. Categorical data were analyzed using the chi-square test. Comparative analyses of descriptive and inferential data were performed using Student's *t-*test or the Mann–Whitney *U*-test. A *p* < 0.05 was considered significant.

### Ethical Approval

The study was approved by the institutional review board of the local institute (CGMH 201901720B0).

## Results

### Demographic Data

A total of 53 cases with clinical suspicion and compatible ultrasonographic findings for liver abscess were collected initially; 11 cases were excluded by CT scan and four cases were excluded due to their inappropriateness for the data analysis: one referral case had incomplete medical records; one case with traumatic abdominal injury had a prolonged and complicated treatment course; and a pair of preterm infants had multiple prematurity-associated complications, a concurrent infection, an uncertain diagnosis and a prolonged antibiotics course. After these exclusions, 38 eligible cases were finally enrolled.

Mean age at diagnosis of the enrolled patients was 9.6 ± 6.2 years (range 1-month to 18-years 6-months) (Table 1). No specific age group with a tendency to develop a liver abscess was identified, although the 10–15-year-old group predominated (Table 1). There were 25 males (66%) and 13 females (34%) (Male-to-female ratio: 1.92:1), and male predominance was noted in three of the four age groups. About half of the patients were diagnosed within the first quarter of the enrollment period (2000–2004, *n* = 18), followed by a gradual declining trend of incidence. Previous Taiwanese pediatric single center-based studies disclosed a decline in incidence from 20 per 100,000 pediatric admissions in 1979–1992 to 8.3 per 100,000 pediatric admissions in 1986–2001. The estimated incidence in the present study was 5.39 per 100,000 pediatric admissions, demonstrating a decreasing trend in Taiwan (8, 9).

**Table 1:** Demographic data, underlying disease, and year of diagnosis.

**Gender (M:F)**		**1.92:1**		
**Age at diagnosis (year)**		**9.6** **±** **6.2**		
	**Age group (year)**	**Male**	**Female**	**Total**
	0–5	8	2	10
	5–10	4	3	7
	10–15	9	3	12
	>15	4	5	9
**Underlying disease**		**Number (%)**		
Hematology-Oncology disease		11 (28.9)		
Hepatobiliary disease		9 (23.7)		
Type I diabetes mellitus		2 (5.3)		
Umbilical venous catheterization		2 (5.3)		
Type II diabetes mellitus		1 (2.6)		
**Year of diagnosis**		**Number (%)**		
2000–2004		18 (47.4)		
2005–2009		7 (18.4)		
2010–2014		7 (18.4)		
2015–2019		6 (15.8)		

### Clinical Manifestations

The leading three symptoms were fever (94.7%), right upper quadrant pain (42.1%), and vomiting (23.7%). Abdominal pain (including right upper quadrant, epigastric, flank, and right lower quadrant) developed in 28 cases (73.7%). Respiratory tract infection symptoms and shortness of breath were documented in nine (23.7%) and six (15.8%) cases, respectively. Pleural effusion, shock, and hyperglycemia occurred in 13 (34.2%), 4 (10.5%), and 3 (7.9%) patients, respectively. Subjective jaundice was observed in two (5.3%) cases (Table 2).

**Table 2:** Clinical presentation.

**Presentation**	**Number (%)**
Fever	36 (94.7)
Abdominal pain (RUQ)	16 (42.1)
Pleural effusion	13 (34.2)
Vomiting	9 (23.7)
Respiratory tract symptom	9 (23.7)
Diarrhea	7 (18.4)
Abdominal pain (Epigastric)	6 (15.8)
Shortness of breath	6 (15.8)
Shock	4 (10.5)
Abdominal pain (Flank)	4 (10.5)
Abdominal pain (RLQ)	2 (5.3)
Body weight loss	2 (5.3)
Jaundice	2 (5.3)

*RUQ, right upper quadrate; RLQ, right lower quadrate*.

Except for two cases, all patients presented initially with fever. The median duration of fever was 19 days (13–25 days). The median durations of fever onset to diagnosis and fever onset to interventional drainage were 9 days (5.5–14.5 days) and 15 days (11–21 days), respectively.

### Underlying Diseases

The most common comorbidities were hematology-oncology disease (28.9%), followed by hepatobiliary disease (23.7%), and diabetes mellitus (7.9%). In the hematology-oncology disease group, four cases were thalassemia, and seven were leukemia. Hepatobiliary disease included biliary atresia, choledochal cysts, hepatic hemochromatosis, hepatic steatosis, and a history of lipid extravasation. Two infantile cases received umbilical venous catheterization. No cases of acute appendicitis, primary immunodeficiadingency, or autoimmune disease were observed.

### Laboratory Findings

Leukocytosis (WBC > 10,000/μL) was noted in 26 cases (70.3%), with a mean of 14,586.5 ± 9643.6/μL. Seven cases (18.4%) had neutropenia (absolute neutrophil count < 1,500/μL) and seven cases (18.4%) had bandemia (band cell > 700/μL). An elevated CRP level (>5 mg/dL) was noted in 37 cases (100%), with a mean of 166.5 ± 106.2 mg/dL. Increased serum levels of alanine aminotransferase, aspartate aminotransferase, direct bilirubin, and total bilirubin were noted in 48.6, 40.5, 23.1, and 42.9% of patients, respectively (Table 3).

**Table 3:** Laboratory examination.

WBC (count/μL) (*n* = 37)	*n* (%)
Mean ± SD	14586.5 ± 9643.6
Leukocytosis (WBC > 10000)	26 (70.3)
Leukopenia (WBC < 4000)	7 (18.9)
Neutropenia (ANC < 1500)	7 (18.9)
Bandemia (Band cell > 700)	7 (18.9)
CRP (mg/dL) (*n* = 37)	*n* (%)
Mean ± SD	166.5 ± 106.2
Elevation (> 5)	37 (100)
Hb (*n* = 38)	*n* (%)
Mean ± SD	10 ± 1.9
Anemia for age	31 (81.6)
AST (*n* = 37)	*n* (%)
Median (IQR)	36 (25–70)
Elevation for age	15 (40.5)
ALT (*n* = 35)	*n* (%)
Median (IQR)	43 (26.5–93.5)
Elevation for age	17 (48.6)
Direct bilirubin (*n* = 26)	*n* (%)
Median (IQR)	0.4 (0.3–1.4)
Elevation for age	6 (23.1)
Indirect bilirubin (*n* = 28)	*n* (%)
Median (IQR)	0.7 (0.5–2.9)
Elevation for age	12 (42.9)

*WBC, white blood cell; ANC, absolute neutrophil count; CRP, c reactive protein; AST, aspartate aminotransferase; ALT, alanine aminotransferase*.

### Diagnostic Approach

#### Imaging Findings

Abdominal ultrasonography was performed in each patient; contrast enhanced CT and MRI were performed in 33 cases (86.8%) and 1 (2.6%) case, respectively. Ten patients (26.3%) had multiple abscesses. Right lobe abscesses, either solitary or multiple, were detected in 29 (76.3%) patients. Seven (18.4%) patients had multiple abscesses involving bilateral lobes. Hepatomegaly was observed in 23 (60.5%) patients, while a concurrent splenic abscess was documented in five (13.2%) patients. The mean size of the abscess (calculated as the longest and maximal diameter for patients with multiple abscesses) was 5.3 ± 3.5 cm (1–15 cm), while 20 (52.6%) and 4 (10.5%) patients had lesions >5 and 10 cm, respectively (Table 4). Liver abscess was incidentally disclosed via a Gallium scan in a case that presented with fever of unknown origin, prolonged dry cough, and respiratory distress.

**Table 4:** Image characteristics of abscess.

**Characteristics**		***n* (%)**
Lesion plurality	Single	28 (73.7)
	Multiple	10 (26.3)
Involved lobe	Right, alone	22 (57.9)
	Left, alone	9 (23.7)
	Bilateral	7 (18.4)
Size (cm)	0–5	18 (47.4)
	>5	20 (52.6)
Splenic abscess		5 (13.2)
Hepatomegaly		23 (60.5)

#### Blood and Abscess Culture Results

All patients received a blood culture, with a 28.9% (*n* = 11) positive culture rate. The most commonly cultured pathogens were *K*. *pneumoniae* (4/11, 36.4%), *Escherichia coli* (2/11, 18.2%), and coagulase negative staphylococci (2/11, 18.2%). Twenty-six (68.4%) patients underwent interventional drainage (percutaneous needle aspiration, pigtail drainage, or surgery); thus, abscess cultures were obtained, with a 53.8% (*n* = 14) positive culture rate. *K*. *pneumoniae* (9/14, 64.3%) and *E*. *coli* (2/14, 14.3%) were the two most common pathogens. Among the antimicrobial resistance profiles of *K*. *pneumoniae* in abscess, one case demonstrated resistance to ampicillin (1/9, 11%); otherwise, there was no specific advanced drug resistant case such as extended spectrum β lactamase (ESBL) producing or *K. pneumoniae* carbapenemase (KPC) producing strain. Polymicrobial growth in the abscess was noted in one case, revealing *E*. *coli, Streptococcus viridans*, and *Peptostreptococcus*. One patient was positive for *K*. *pneumoniae* in both the blood and abscess cultures (Table 5). Among the four cases that were complicated with septic shock, two were confirmed to have *Klebsiella* sp.-associated bacteremia. Tuberculosis (TB)-related liver abscesses were documented in two cases; one underwent surgical drainage to obtain supportive pathological evidence describing a granulomatous change; the other manifested as congenital disseminated TB with multiple hepatosplenic abscesses. Patients with underlying leukemia were administered an empirical antifungal agent because of clinical suspicion of a fungal abscess.

**Table 5:** Blood and abscess culture results.

**Blood culture**	**%**
Blood culture rate	100 (38/38)
Blood culture positive rate	28.9 (11/38)
*Klebsiella pneumonia*	36.4 (4/11)
*Escherichia coli*	18.2 (2/11)
CONS	18.2 (2/11)
*Klebsiella oxytoca*	9 (1/11)
*Pseudomonas aeruginosa*	9 (1/11)
*Viridans streptococcus*	9 (1/11)
**Abscess culture**^†^	**%**
Abscess culture rate	68.4 (26/38)
Abscess culture positive rate	53.9 (14/26)
*Klebsiella pneumonia*	64.3 (9/14)
*Escherichia coli*	14.3 (2/14)
Viridans streptococcus/Streptococcus-like	7.1 (1/14)
*Bacteroides thetaiotaomicron*	7.1 (1/14)
*Peptostreptococcus micros*	7.1 (1/14)
*Enterococcus faecium*	7.1 (1/14)
*Staphylococcus aureus*	7.1 (1/14)

*CONS, coagulase negative staphylococci. A patient yielded three microbes*.

### Treatment and Outcome

Antimicrobial therapy was administered to all patients, adjusted by the clinical response and available culture results. The empirical broad-spectrum regimen applied in the present study was comprised of a cephalosporin, an aminoglycoside, and an anti-anaerobic agent. Six cases receiving a prolonged course of medication for suspected/confirmed fungal or mycobacterial-related abscesses were excluded from the statistical analysis of medical treatment duration. The mean duration of parenteral antibiotic therapy was 29 ± 15.7 days (10–77 days) and the mean duration of total antibiotic therapy was 45.1 ± 22.1 days (14–103 days). An exclusive antibiotic treatment was documented in 12 cases (31.6%) (Table 6).

**Table 6:** Treatment and comparison of characteristic between drainage and non-drainage group.

**Type of therapy**	***n* (%)**	**Duration (day), median (IQR)**	
Needle aspiration	12 (31.6)		
Pigtail drainage	10 (26.3)	10.5 (6.25–15)	
Surgical drainage	5 (13.2)		
Antibiotics alone	12 (31.6)		
Antibiotics duration	Regimen	Day, Mean ± SD^†^	
	Parenteral	29 ± 15.7	
	Parenteral + Oral	45.1 ± 22.1	
	**Drainage**	**Non-drainage**	***p*****-value**
Age (year)	9.6 ± 5.4	9.3 ± 7.9	> 0.05
Gender (M:F)	1.4:1	5:1	> 0.05
WBC (mean)	15412	12866.7	> 0.05
CRP (mean)	178.8	137.3	> 0.05
AST (mean)	60.7	122.1	> 0.05
ALT (mean)	67.7	106.1	> 0.05
Abscess size (cm) (mean)	6.5	2.7	0.00011*
Febrile duration (day) (mean)	19.5	22.2	> 0.05

WBC, white blood cell; CRP, c reactive protein; AST, aspartate aminotransferase; ALT, alanine aminotransferase. Exclusion of cases with extremely prolonged course with suspicion of fungal/mycobacterial infection. *p < 0.05.

Interventional drainage, including image-guided needle aspiration, pigtail drainage, and surgery, were performed on 12 (31.6%), 10 (26.3%), and 5 (13.2%) cases, respectively, with a median duration of fever onset to drainage of 15 days (11–21 days). Needle aspiration was assisted by ultrasonographic guidance and a CT scan in a few cases. One patient received an exploratory operation and intraoperative placement of a pigtail for continuous drainage. Mean duration of pigtail placement was 15.2 days (4–54 days), while the median duration was 10.5 days (6.25–15 days). The characteristics and initial laboratory results between the drainage and non-drainage group were compared in Table 6. The size of the abscesses was significantly larger in the drainage group than in the non-drainage group. However, no significant differences were observed in age, gender, liver enzyme level, or total fever duration. Patients in the drainage group presented with higher WBC and CRP levels, but the trend was not significant. Five patients (13.2%) diagnosed before 2007 underwent surgical drainage, including a patient with a solitary abscess reaching 15 cm in size, and two patients with a comorbid para-splenic abscess and profound empyema required a concurrent decorticative operation, respectively

Three patients developed a recurrent abscess, including one patient with biliary atresia and a history of recurrent biliary tract infection, one asplenic patient with Cooley's anemia, and another patient with leukemia and a concurrent splenic abscess. No abscess rupture, peritonitis, or abscess-associated mortality was observed within the follow up period, except the four cases complicated with shock.

## Discussion

The present study reported 38 pediatric patients with liver abscesses in a 20-year period. The estimated incidence in the present study was 5.39 per 100,000 pediatric admissions during 2011–2018. Similar to previous studies, a declining trend of incidence was observed among Taiwanese children, even though those previous studies were not population-based. Incidence varies among studies from different countries, reflecting a possible association with sanitation, healthcare access, and nutritional condition (2, 3, 14, 15).

The mean age at diagnosis was 9.6 ± 6.2 years in the present study, with 55.2% (21/38) of patients aged > 10 years old, which was a lower proportion compared to two previous single center-based studies in Taiwan (86.7% [13/15] in 2003 and 86.7% [13/15] in 2015) (9, 10). Age at diagnosis reported in most studies was close, including 10 years in a UK study and 8 years in an Iranian study (14, 16). A few studies conducted in the developing world disclosed a trend of a younger age at diagnosis, such as 6.3 years in west India, 8.1 years in Brazil, and 5.4 years in the Côte d'Ivoire (3, 15, 17). Earlier and prolonged exposures to a pathogenic source with limited hygiene in these tropical or developing areas were proposed as reasons contributing to this trend of younger occurrence (3, 14). Male predominance (66%) was observed in the present study, this was similar to studies conducted in the Côte-d'Ivoire (male, 60%) and Brazil (male, 72%), while studies from India (male, 44%), the UK (male, 46%), and a previous Taiwanese study (male, 40%) showed female predominance.

The most prevalent symptoms of fever and abdominal pain were similar to most previous reports (2, 9, 10, 14). Prolonged fever commonly developed, as the median febrile duration before diagnosis exceeded 7 days. The location of pain was diverse, although the right upper quadrant was the most common. Pleural effusion, respiratory tract symptoms, and shortness of breath developed in a considerable percentage of patients, which could possibly be explained by subdiaphragmatic irritation or pleuropulmonary spread of the liver abscess (2, 10). Although jaundice has been revealed as characteristic of the high-risk group, it has been relatively less discussed in pediatric studies (18). The incidence of jaundice in pediatric series reported from northern Taiwan and New Delhi were 33 and 18.8%, respectively (4). In the present study, subjective jaundice developed in only two cases and was poorly correlated with actual laboratory hyperbilirubinemia. Overall, the initial presentations were mostly non-specific, while <40% of patients fulfilled Fontan's triad (fever, right hypochondrium pain, and hepatomegaly).

Underlying disease and predisposing host factors that have been proposed to be associated with the development of liver abscess include immunocompromised status (malignancy, malnutrition, and chemotherapy or immunosuppressant user), chronic granulomatous disease, sickle cell disease, diabetes mellitus, biliary tract anomalies, abdominal trauma, certain parasitic infections, systemic sepsis, perforated appendicitis, umbilical infection, and incorrect umbilical venous catheterization (2, 10, 15, 16, 19–22). Nevertheless, the majority of pyogenic liver abscesses are cryptogenic (2, 23). Cryptogenic liver abscess accounted for 47.4% (18/38) of cases in our study, which was a comparatively lower proportion to prior Taiwanese reports among children (53–80%) (8–10). About 25% of patients carried hepatobiliary diseases with or without enterobiliary manipulation, while 55% (5/9) underwent portoenterostomy (24). Hematologic malignancy, such as acute leukemia, conveys an increased risk for a visceral abscess, which has been ascribed to cytotoxic therapy and subsequent neutropenia. In addition, these patients carry a higher risk of fungal infection (25). Hepatic infection has been associated with morbidity and mortality in children receiving chemotherapy, yet the non-specific manifestations and non-standardized diagnosis trouble clinicians (26). Among the seven enrolled cases of leukemia, all encountered neutropenic fever prior to the abscess diagnosis. Bacterial cultures were positive in three, while six cases were treated with suspicion of a fungal infection. Two cases developed a disseminated hepatosplenic abscess. Overall, they tended to receive prolonged and broad-spectrum antimicrobial therapy due to a prolonged febrile period, difficult clinical judgment, and concern for their immunocompromised status. Liver abscesses have been reported in adults and children with thalassemia, with or without transfusion dependence (27–29). One of the four cases of thalassemia received a splenectomy, which has been regarded as a risk factor for development of a liver abscess (28, 30). Diabetes mellitus, a potential negative factor for the prognosis of liver abscess, was also documented in three cases, and the causal pathogen was *K*. *pneumoniae* in all cases (7, 31–33). Although primary immunodeficiency and perforated appendicitis were referred as possible risk factors, we reported no patients in these categories.

Abnormal laboratory parameters involved in cases of liver abscess discussed in the literature include leukocytosis, anemia, and elevated acute phase reactants (erythrocyte sedimentation rate, CRP, and altered liver enzyme levels) (2). However, the correlation between these abnormalities and severity was less obvious and none of them was specific. In our series, all patients presented with an elevated CRP level, yet abnormal liver enzymes or hyperbilirubinemia were found in less than half, which paralleled the relatively scarce occurrence of clinical subjective jaundice. An Indian study (*n* = 34) described no abnormal liver function tests and no one suffered from clinical icterus (17). Among patients encountering the chemotherapy-related neutropenic phase, leukocytosis was even less valuable as a laboratory predictor, while neutropenia has been documented as a predictor.

Our study demonstrated a positive abscess culture rate of 53.8% (14/26) and a positive blood culture rate of 28.9% (11/38). The positive abscess culture rates were 19.2% (5/26), 23.5% (8/34), 50% (8/16), 67% (10/15), 73/3% (11/15), and 85.7% (12/14) in studies from the Côte-d'Ivoire, India, Iran, UK, southern Taiwan, and central Taiwan, respectively. The positive blood culture rate appeared lower in general compared to previous studies, such as 13.3% (2/15) and 20% (3/15) in southern and central Taiwan (3, 9, 10, 14, 16, 17). We reported no amebic or culture-proved fungal abscesses. Amebic liver abscesses (ALAs) are uncommon in children, with occurrence influenced mostly by endemic factors (17). The reported incidence of ALAs for children varies, such as 7% (1/15), 30% (9/30), 35% (9/26), and 50% (10/20) from studies in Taiwan, Côte-d'Ivoire, Senegal, and Spain (3, 9, 19, 34). Although *S. aureus* is the leading causal pathogen in the majority of pediatric reports, the present study reports the dominance of *K*. *pneumoniae*, paralleling three Taiwanese pediatric series (8–10, 14). The second most common pathogen was *E*. *coli* in blood and abscess cultures. *S*. *aureus* was detected in only one case. Hepatic TB is uncommon, while a TB liver abscess is even rarer. Hepatic TB accounts for 1.2% of all TB cases and TB liver abscesses develop in 0.34% of hepatic TB cases (35). Overall, about 25 cases of isolated TB liver abscess have been reported in the English literature before 2003, while pediatric cases remain scarce (35–38). The diagnosis of a TB abscess remains challenging because of the lack of a specified presentation and the limited yield of tests.

Abdominal ultrasonography was performed in all patients in our series, but a few cases were excluded because of a bilioma, cyst, or hepatic metastases on CT or MRI. Chuang et al. observed that the sensitivity of ultrasonography ranges from 66 to 100%, yet the false positive rate could reach 55.6% despite typical imaging features and a well-trained operator (13). Nevertheless, ultrasonography is still recommended as the mainstay screening modality owing to lower cost, lack of radiation, and non-invasive properties (3). The right-lobe and solitary-lesion preponderance observed in our cases resembled prior studies, except a UK study found that 86.6% (13/15) of cases developed multiple or multiloculated abscesses (2, 9, 14, 17, 19). The mean size of the largest diameter abcess (5.3 ± 3.5 cm) among our cases was smaller, compared with prior reports in southern Taiwan (6.9), central Taiwan (6.6), and Côte-d'Ivoire (11.8) (3, 9, 10).

Antibiotic therapy contributes to a crucial part of management. The proportion of exclusive medical therapy was 31.6% (12/38), appearing higher than most prior experiences, and reported as 7% (1/15), 7% (1/15), 9% (3/34), and 13% (4/30) in central Taiwan, southern Taiwan, Côte-d'Ivoire, and India, respectively (3, 7, 10, 17). A Spanish study suggested that 80% of patients eventually require aspirational intervention (34). However, our results were closer to those proposed by a South African study, which revealed a 37% success rate with antibiotic therapy alone (39). The duration of antibiotic therapy in our cases generally paralleled the suggested strategies in previous studies, such as 2–4 weeks of parenteral antibiotics followed by oral antibiotics to complete a 4–6 week course (2, 40).

Percutaneous drainage (PD) benefited our patients by evacuating pus and providing microbial information. The mean duration of pigtail placement in our series (mean, 15.2 days) was close to a prior report in southern Taiwan (mean, 13.1 days), but almost double that of an Indian study (mean, 7.7 days) (9, 17). Nevertheless, we report no PD-associated complications, including bacteremia, hemobilia, hemoperitoneum, or iatrogenic infection (2). The success rate of PD is as high as 92% in one study, while the failure rate is 5–30.8% and is affected by lesion characteristics (17, 18). PD may enhance the safety and efficacy of treating abscesses.

Although Bari et al. regarded open surgical drainage as the best treatment modality while utilizing a surgical approach in 42.6% (55/129) of their patients, we tended to follow the strategy of preserving surgical intervention for cases of PD failure, concurrent intra-abdominal pathology, or a ruptured or large abscess (40, 41). Apart from the advocated advantages of surgical drainage, complications including an intestinal adhesion obstruction, fistula, and incisional hernia have been observed in a pediatric series (39). The ideal cut-off size of an abscess to decide between percutaneous or surgical drainage remains controversial, even in adults (42, 43). In addition, conditions such as multiple abscesses and chronic granulomatous disease require additional evaluation or even individualized preoperative considerations (2, 44). Thus, optimizing surgical drainage for pediatric patients awaits further comprehensive evidence.

The mortality rate from pediatric liver abscess has been reduced from 42% to <15% or even null in recent studies through advances in diagnostic and therapeutic approaches (2, 3, 10, 14, 40). However, intensive care was required for patients presenting with severe septic shock or clinical complications. Prognostic determinants of complications suggested in a Mexican adult study included abdominal pain and tachypnea, alanine aminotransferase >154 IU/L, hemoglobin <10 g/dL, and a ruptured abscess. Other adult studies have also proposed that major underlying disease (malignancy and severe organ dysfunction), gas-forming abscesses, multi-drug-resistant isolates, anaerobic infection, high blood urea nitrogen, and a high Acute Physiology and Chronic Health Evaluation II score at admission are the main prognostic factors for mortality (40, 45, 46). However, a pediatric stratification system is still lacking.

Bias from subjective symptoms and variations in the physician's clinical judgment may have confounded the data collection in our study. In addition, missing laboratory data and incomplete chart records limited the extent of our retrospective analysis. Nevertheless, the present study provides the largest pediatric series in the last two decades, demonstrating updated, regional data, including demographics, diagnostic approaches, therapeutic preferences, and outcomes, in an attempt to aid in advancing future management.

## Conclusion

Pediatric liver abscess is uncommon yet its identification and timely diagnosis require a high index of suspicion, particularly in susceptible children with a history of hepatobiliary, hematological disease, and malignancy. The present study reflects a relatively declining incidence of liver abscess compared with prior studies in Taiwan. *K*. *pneumoniae* remained the most prevalent pathogen in both blood and abscess cultures, which agrees with previous Taiwanese studies. Combined therapy comprised of antibiotics and drainage was the mainstay in our series, yet 31.6% (12/38) of patients received exclusive medical therapy alone. Proper antimicrobial therapy with timely drainage generally yielded an adequate treatment response without any mortality.

## Data Availability Statement

All datasets generated for this study are included in the article/supplementary material.

## Ethics Statement

The studies involving human participants were reviewed and approved by the institutional review board of Chang Gung Memorial Hospital, IRB number 201901720B0. Written informed consent from the participants' legal guardian/next of kin was not required to participate in this study in accordance with the national legislation and the institutional requirements. Written informed consent was not obtained from the minor(s)' legal guardian/next of kin for the publication of any potentially identifiable images or data included in this article.

## Author Contributions

C-CC, H-YY, M-WL, and H-CC: diagnosis and management of the patients. P-JY and H-CC: writing of the manuscript, data analysis and interpretation, and final approval of the manuscript. H-CC critical evaluation and revision of the manuscript.

### Conflict of Interest

The authors declare that the research was conducted in the absence of any commercial or financial relationships that could be construed as a potential conflict of interest.
